# 5,7-Dihy­droxy-2-(3-hy­droxy-4,5-dimeth­oxy­phen­yl)-6-meth­oxy-4*H*-chromen-4-one

**DOI:** 10.1107/S1600536813007381

**Published:** 2013-03-23

**Authors:** Shahobiddin M. Adizov, Rimma F. Mukhamathanova, Kambarali K. Turgunov, Ildar D. Sham’yanov, Bakhodir Tashkhodjaev

**Affiliations:** a S. Yunusov Institute of the Chemistry of Plant Substances, Academy of Sciences of Uzbekistan, Mirzo Ulugbek Str. 77, Tashkent 100170, Uzbekistan

## Abstract

The title compound, C_18_H_16_O_8_, was isolated from the plant *Artemisia baldshuanica* Krasch et Zarp. The mol­ecule is approximately planar, with the exception of the terminal methyl groups, the C atoms of which devitate from their attached ring planes by 1.243 (5) and 1.168 (5) Å. The dihedral angle between the substituted benzopyran and benzene rings is 5.8 (1)°; this near planarity could be due to conjugation or a packing effect. Intra­molecular O—H⋯O and C—H⋯O hydrogen bonds occur. In the crystal, mol­ecules are connected by O—H⋯O hydrogen bonds involving the hy­droxy and carbonyl groups, forming hydrogen-bonded chains along [001] and [1-10]. The chains are linked by C—H⋯O inter­actions.

## Related literature

For the biological activity of flavonoids, see: Bodewes *et al.* (2011 ▶); Veitch & Grayer (2011 ▶). For related structures, see: Martinez-Vazquez *et al.* (1993 ▶).
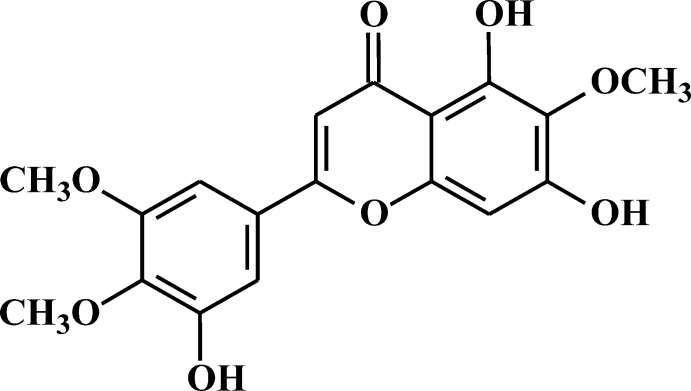


## Experimental

### 

#### Crystal data

C_18_H_16_O_8_
*M* *_r_* = 360.31
Monoclinic, 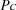

*a* = 11.5837 (6) Å
*b* = 4.4677 (3) Å
*c* = 15.5521 (8) Å
β = 103.310 (6)°
*V* = 783.23 (8) Å^3^
*Z* = 2
Cu *K*α radiation
μ = 1.04 mm^−1^
*T* = 300 K
0.60 × 0.40 × 0.20 mm

#### Data collection

Oxford Diffraction Xcalibur Ruby diffractometer
Absorption correction: multi-scan (*CrysAlis PRO*; Oxford Diffraction, 2009 ▶) *T*_min_ = 0.638, *T*_max_ = 0.813
2540 measured reflections
1841 independent reflections
1632 reflections with *I* > 2σ(*I*)
*R*_int_ = 0.018

#### Refinement

*R*[*F* ^2^ > 2σ(*F* ^2^)] = 0.042
*wR*(*F* ^2^) = 0.126
*S* = 1.07
1841 reflections
242 parameters
2 restraints
H-atom parameters constrained
Δρ_max_ = 0.16 e Å^−3^
Δρ_min_ = −0.19 e Å^−3^

### 

Data collection: *CrysAlis PRO* (Oxford Diffraction, 2009 ▶); cell refinement: *CrysAlis PRO*; data reduction: *CrysAlis PRO*; program(s) used to solve structure: *SHELXS97* (Sheldrick, 2008 ▶); program(s) used to refine structure: *SHELXL97* (Sheldrick, 2008 ▶); molecular graphics: *XP* in *SHELXTL* (Sheldrick, 2008 ▶); software used to prepare material for publication: *publCIF* (Westrip, 2010 ▶).

## Supplementary Material

Crystal structure: contains datablock(s) I, global. DOI: 10.1107/S1600536813007381/zp2001sup1.cif

Structure factors: contains datablock(s) I. DOI: 10.1107/S1600536813007381/zp2001Isup2.hkl

Supplementary material file. DOI: 10.1107/S1600536813007381/zp2001Isup3.cml

Additional supplementary materials:  crystallographic information; 3D view; checkCIF report

## Figures and Tables

**Table 1:** Hydrogen-bond geometry (Å, °)

*D*—H⋯*A*	*D*—H	H⋯*A*	*D*⋯*A*	*D*—H⋯*A*
O2—H2*A*⋯O3	0.82	2.38	2.762 (4)	109
O2—H2*A*⋯O5^i^	0.82	1.96	2.713 (4)	152
O4—H4*A*⋯O5	0.82	1.85	2.579 (4)	148
O6—H6*A*⋯O4^ii^	0.82	2.20	2.954 (3)	153
O6—H6*A*⋯O7	0.82	2.31	2.734 (4)	113
C3—H3*A*⋯O2^iii^	0.93	2.58	3.246 (5)	129
C12—H12*B*⋯O4^iv^	0.96	2.55	3.459 (6)	157
C12—H12*C*⋯O8	0.96	2.32	2.927 (6)	122

Symmetry codes: (i) 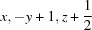; (ii) 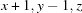; (iii) 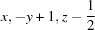; (iv) 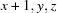.

**Fig. 1.**
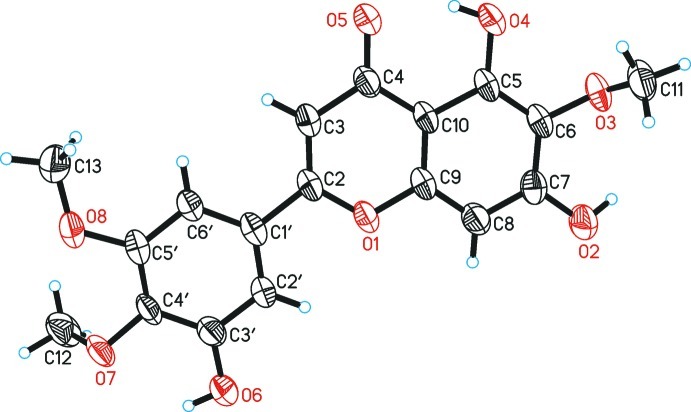
Molecular structure of the title compound, displacement ellipsoids are drawn at the 50% probability level.

## supplementary crystallographic information

### Comment

Among of biologically active natural compounds of plant origin flavonoids occupy an important place. Most important for practical medicine are flavonoids with antioxidant, antiinflammatory, antispasmodic and anticancer activity (Bodewes *et al.*, 2011; Veitch *et al.*, 2011).

*Artemisia baldshuanica Krasch. et Zarp*. (*Asteraceae*) - is a widespread in centralasia species of Artemisia and juice of leaves possesses a antihelmintic activity. The title compound, 5,7,3'-trihydroxy-6,4',5'-trimethoxyflavone (also named as 5,7,-dihydroxy-2-(3-hydroxy-4,5-dimethoxyphenyl)-6-methoxy-4*H*- chromen-4-one) was isolated from the ethanol extract of above-ground part of *Artemisia baldshuanica*.

The molecule is nearly planar with exception of H_3_C11- and H_3_C12- terminal methyl groups. Substituted benzopyran and phenyl rings are planar with r.m.s. deviations of 0.020 Å and 0.026 Å, respectivily. The angle between ring planes equals to 5.8 (1)° thanks to conjugation of π-electronic systems of cycles. Terminal methyl group carbon atoms (C11 and C12) are deviated from their ring planes to 1.243 (5) Å and 1.168 (5) Å, respectively.

The molecule has intramolecular hydrogen bond where O4-H4A···O5 hydroxylic and ketonic groups form six membered pseudocycle. (Table 1). Also it are observed O2-H2A···O3-CH_3_ and O6-H6A···O7-CH_3_ intramolecular hydrogen bonds between hydroxyl and methoxyl groups. In the crystal, molecules are interconnected *via* O2-H2A···O5 and O6-H6A···O4 hydrogen bonds observed between hydroxyl and carbonyl groups forming three dimensional H-bond chains. The molecules are further linked by a weak C2'-H2'A···O1, C3-H3A···O2, C12-H12B···O4 and C12-H12C···O8 intermolecular interactions (Table 1).

### Experimental

Air dry powdered aerial part of Artemisia baldshuanica, collected prebudding period four time extracted with ethanol. The combined extracts were concentrated under vacuum on a rotary evaporator. Concentrated extract was fractionated with benzene, chloroform, ethylacetate and ethanol. Chloroformic fraction was chromatographed on a silica gel column. Eluting the column with chloroform-ethanol (50:1) was isolated dark-yellow crystals with m.p 244-245 ° C. Crystals suitable for X-ray diffraction were obtained by slow evaporation of a solution of the compound in ethanol.

### Refinement

All H atoms were placed geometrically and treated as riding on their parent atoms with C–H = 0.96 Å (methyl) or 0.93 Å (aromatic) and O-H=0.82 Å with *U*_iso_(H) = 1.5Ueq(C) (methyl), *U*_iso_(H) = 1.2Ueq(C) (aromatic) and *U*_iso_(H) = 1.5Ueq(O). In the absence of significant anomalous scattering effects Friedel pairs have been merged.

### Crystal data

**Table:**

C_18_H_16_O_8_	*F*(000) = 376
*M**_r_* = 360.31	*D*_x_ = 1.528 Mg m^−^^3^
Monoclinic, *P**c*	Melting point: 517(1) K
Hall symbol: P -2yc	Cu *K*α radiation, λ = 1.54184 Å
*a* = 11.5837 (6) Å	Cell parameters from 1516 reflections
*b* = 4.4677 (3) Å	θ = 3.9–75.1°
*c* = 15.5521 (8) Å	µ = 1.04 mm^−^^1^
β = 103.310 (6)°	*T* = 300 K
*V* = 783.23 (8) Å^3^	Prismatic, yellow
*Z* = 2	0.60 × 0.40 × 0.20 mm

### Data collection

**Table:**

Oxford Diffraction Xcalibur Ruby diffractometer	1841 independent reflections
Radiation source: Enhance (Cu) X-ray Source	1632 reflections with *I* > 2σ(*I*)
Graphite monochromator	*R*_int_ = 0.018
Detector resolution: 10.2576 pixels mm^-1^	θ_max_ = 72.0°, θ_min_ = 3.9°
ω scans	*h* = −11→14
Absorption correction: multi-scan (*CrysAlis PRO*; Oxford Diffraction, 2009)	*k* = −3→5
*T*_min_ = 0.638, *T*_max_ = 0.813	*l* = −19→19
2540 measured reflections	

### Refinement

**Table:**

Refinement on *F*^2^	Secondary atom site location: difference Fourier map
Least-squares matrix: full	Hydrogen site location: inferred from neighbouring sites
*R*[*F*^2^ > 2σ(*F*^2^)] = 0.042	H-atom parameters constrained
*wR*(*F*^2^) = 0.126	*w* = 1/[σ^2^(*F*_o_^2^) + (0.0802*P*)^2^ + 0.1369*P*] where *P* = (*F*_o_^2^ + 2*F*_c_^2^)/3
*S* = 1.07	(Δ/σ)_max_ = 0.001
1841 reflections	Δρ_max_ = 0.16 e Å^−^^3^
242 parameters	Δρ_min_ = −0.19 e Å^−^^3^
2 restraints	Extinction correction: *SHELXL97* (Sheldrick, 2008), Fc^*^=kFc[1+0.001xFc^2^λ^3^/sin(2θ)]^-1/4^
Primary atom site location: structure-invariant direct methods	Extinction coefficient: 0.008 (2)

### Special details

**Table:**

Geometry. All e.s.d.'s (except the e.s.d. in the dihedral angle between two l.s. planes) are estimated using the full covariance matrix. The cell e.s.d.'s are taken into account individually in the estimation of e.s.d.'s in distances, angles and torsion angles; correlations between e.s.d.'s in cell parameters are only used when they are defined by crystal symmetry. An approximate (isotropic) treatment of cell e.s.d.'s is used for estimating e.s.d.'s involving l.s. planes.
Refinement. Refinement of *F*^2^ against ALL reflections. The weighted *R*-factor *wR* and goodness of fit *S* are based on *F*^2^, conventional *R*-factors *R* are based on *F*, with *F* set to zero for negative *F*^2^. The threshold expression of *F*^2^ > σ(*F*^2^) is used only for calculating *R*-factors(gt) *etc*. and is not relevant to the choice of reflections for refinement. *R*-factors based on *F*^2^ are statistically about twice as large as those based on *F*, and *R*- factors based on ALL data will be even larger.

### Fractional atomic coordinates and isotropic or equivalent isotropic displacement parameters (Å^2^)

**Table:**

	*x*	*y*	*z*	*U*_iso_*/*U*_eq_	
O1	0.4704 (2)	0.1567 (6)	0.32058 (17)	0.0446 (6)	
O5	0.2167 (2)	0.7371 (7)	0.19407 (19)	0.0537 (7)	
O4	0.1091 (2)	0.6830 (6)	0.32115 (18)	0.0499 (6)	
H4A	0.1203	0.7391	0.2735	0.075*	
O3	0.1066 (2)	0.4181 (6)	0.48320 (16)	0.0507 (7)	
O2	0.2852 (2)	0.0185 (7)	0.55401 (18)	0.0544 (7)	
H2A	0.2481	0.1197	0.5825	0.082*	
C2	0.4798 (3)	0.2888 (8)	0.2441 (2)	0.0391 (7)	
C3	0.3979 (3)	0.4806 (9)	0.2006 (2)	0.0443 (8)	
H3A	0.4071	0.5636	0.1478	0.053*	
C4	0.2961 (3)	0.5607 (8)	0.2339 (2)	0.0405 (7)	
C5	0.1957 (3)	0.4917 (8)	0.3591 (2)	0.0395 (7)	
C6	0.1942 (3)	0.3556 (8)	0.4387 (2)	0.0412 (7)	
C7	0.2850 (3)	0.1563 (8)	0.4774 (2)	0.0426 (8)	
C8	0.3774 (3)	0.0899 (9)	0.4375 (2)	0.0454 (8)	
H8A	0.4373	−0.0424	0.4635	0.054*	
C9	0.3774 (3)	0.2264 (8)	0.3583 (2)	0.0385 (7)	
C10	0.2897 (3)	0.4242 (8)	0.3162 (2)	0.0378 (7)	
O8	0.7492 (3)	0.3071 (7)	0.03979 (19)	0.0587 (8)	
O7	0.9075 (2)	−0.0387 (6)	0.1554 (2)	0.0518 (7)	
O6	0.8517 (2)	−0.2462 (7)	0.3062 (2)	0.0561 (7)	
H6A	0.9159	−0.2482	0.2925	0.084*	
C1'	0.5891 (3)	0.1945 (8)	0.2175 (2)	0.0397 (7)	
C2'	0.6697 (3)	0.0105 (8)	0.2724 (2)	0.0420 (8)	
H2'A	0.6530	−0.0633	0.3241	0.050*	
C3'	0.7758 (3)	−0.0643 (8)	0.2503 (2)	0.0432 (8)	
C4'	0.8016 (3)	0.0457 (8)	0.1735 (2)	0.0413 (8)	
C5'	0.7192 (3)	0.2219 (9)	0.1170 (2)	0.0443 (8)	
C6'	0.6126 (3)	0.3017 (8)	0.1387 (2)	0.0445 (8)	
H6'A	0.5582	0.4241	0.1014	0.053*	
C11	0.0019 (4)	0.2442 (10)	0.4528 (3)	0.0587 (10)	
H11A	−0.0520	0.2790	0.4903	0.088*	
H11B	0.0225	0.0358	0.4544	0.088*	
H11C	−0.0351	0.3008	0.3933	0.088*	
C12	0.9874 (4)	0.2038 (10)	0.1495 (4)	0.0619 (11)	
H12A	1.0519	0.1304	0.1262	0.093*	
H12B	1.0180	0.2864	0.2073	0.093*	
H12C	0.9455	0.3563	0.1112	0.093*	
C13	0.6770 (4)	0.5095 (10)	−0.0170 (3)	0.0563 (10)	
H13A	0.7116	0.5524	−0.0661	0.085*	
H13B	0.6701	0.6913	0.0143	0.085*	
H13C	0.5997	0.4236	−0.0382	0.085*	

### Atomic displacement parameters (Å^2^)

**Table:**

	*U*^11^	*U*^22^	*U*^33^	*U*^12^	*U*^13^	*U*^23^
O1	0.0371 (12)	0.0586 (14)	0.0446 (12)	0.0102 (11)	0.0227 (10)	0.0024 (11)
O5	0.0449 (14)	0.0703 (16)	0.0533 (14)	0.0189 (13)	0.0266 (12)	0.0137 (13)
O4	0.0382 (12)	0.0658 (15)	0.0526 (14)	0.0148 (12)	0.0246 (11)	0.0045 (12)
O3	0.0449 (13)	0.0656 (15)	0.0509 (15)	0.0012 (13)	0.0304 (11)	−0.0103 (12)
O2	0.0553 (16)	0.0686 (17)	0.0462 (14)	0.0087 (14)	0.0258 (12)	0.0061 (12)
C2	0.0319 (16)	0.0484 (17)	0.0413 (17)	0.0022 (14)	0.0170 (13)	−0.0078 (14)
C3	0.0415 (17)	0.055 (2)	0.0429 (18)	0.0064 (17)	0.0233 (15)	0.0006 (15)
C4	0.0316 (15)	0.0505 (18)	0.0423 (18)	0.0038 (15)	0.0146 (14)	−0.0026 (15)
C5	0.0321 (15)	0.0503 (17)	0.0409 (16)	0.0003 (14)	0.0183 (13)	−0.0065 (14)
C6	0.0329 (16)	0.0533 (17)	0.0421 (16)	0.0009 (14)	0.0184 (13)	−0.0092 (15)
C7	0.0464 (19)	0.0498 (17)	0.0359 (17)	−0.0032 (16)	0.0181 (14)	−0.0067 (14)
C8	0.0381 (17)	0.0547 (19)	0.0459 (18)	0.0058 (15)	0.0147 (14)	−0.0003 (16)
C9	0.0336 (15)	0.0479 (17)	0.0383 (16)	0.0012 (14)	0.0170 (13)	−0.0071 (13)
C10	0.0319 (15)	0.0469 (16)	0.0390 (16)	0.0024 (13)	0.0171 (13)	−0.0069 (13)
O8	0.0532 (16)	0.082 (2)	0.0504 (15)	0.0173 (15)	0.0314 (13)	0.0131 (14)
O7	0.0425 (14)	0.0519 (14)	0.0716 (18)	0.0116 (12)	0.0354 (13)	0.0020 (13)
O6	0.0440 (14)	0.0678 (16)	0.0636 (16)	0.0180 (13)	0.0272 (12)	0.0145 (14)
C1'	0.0313 (15)	0.0485 (17)	0.0437 (17)	−0.0019 (14)	0.0175 (13)	−0.0074 (14)
C2'	0.0397 (17)	0.0483 (17)	0.0439 (18)	0.0043 (15)	0.0219 (15)	0.0025 (14)
C3'	0.0368 (17)	0.0447 (17)	0.0509 (19)	0.0060 (14)	0.0155 (15)	−0.0016 (15)
C4'	0.0335 (16)	0.0448 (16)	0.0529 (19)	0.0055 (13)	0.0250 (15)	−0.0057 (15)
C5'	0.0423 (18)	0.0553 (19)	0.0421 (18)	0.0015 (15)	0.0235 (15)	−0.0037 (15)
C6'	0.0370 (16)	0.0566 (19)	0.0445 (18)	0.0085 (16)	0.0188 (15)	0.0026 (15)
C11	0.042 (2)	0.076 (3)	0.065 (2)	−0.0034 (18)	0.0269 (18)	0.001 (2)
C12	0.0394 (19)	0.060 (2)	0.093 (3)	0.0001 (17)	0.029 (2)	0.002 (2)
C13	0.056 (2)	0.066 (2)	0.051 (2)	−0.0023 (19)	0.0207 (18)	0.0105 (18)

### Geometric parameters (Å, º)

**Table:**

O1—C2	1.354 (4)	O8—C13	1.399 (5)
O1—C9	1.376 (4)	O7—C4'	1.373 (4)
O5—C4	1.260 (4)	O7—C12	1.441 (5)
O4—C5	1.346 (4)	O6—C3'	1.355 (5)
O4—H4A	0.8200	O6—H6A	0.8200
O3—C6	1.382 (4)	C1'—C2'	1.382 (5)
O3—C11	1.425 (5)	C1'—C6'	1.399 (5)
O2—C7	1.341 (4)	C2'—C3'	1.391 (5)
O2—H2A	0.8200	C2'—H2'A	0.9300
C2—C3	1.340 (5)	C3'—C4'	1.387 (5)
C2—C1'	1.482 (4)	C4'—C5'	1.385 (5)
C3—C4	1.438 (4)	C5'—C6'	1.399 (5)
C3—H3A	0.9300	C6'—H6'A	0.9300
C4—C10	1.436 (5)	C11—H11A	0.9600
C5—C6	1.383 (5)	C11—H11B	0.9600
C5—C10	1.434 (4)	C11—H11C	0.9600
C6—C7	1.403 (5)	C12—H12A	0.9600
C7—C8	1.387 (5)	C12—H12B	0.9600
C8—C9	1.374 (5)	C12—H12C	0.9600
C8—H8A	0.9300	C13—H13A	0.9600
C9—C10	1.391 (5)	C13—H13B	0.9600
O8—C5'	1.378 (4)	C13—H13C	0.9600
			
C2—O1—C9	120.3 (3)	C2'—C1'—C2	119.7 (3)
C5—O4—H4A	109.5	C6'—C1'—C2	119.9 (3)
C6—O3—C11	113.0 (3)	C1'—C2'—C3'	120.0 (3)
C7—O2—H2A	109.5	C1'—C2'—H2'A	120.0
C3—C2—O1	122.1 (3)	C3'—C2'—H2'A	120.0
C3—C2—C1'	126.3 (3)	O6—C3'—C4'	121.9 (3)
O1—C2—C1'	111.6 (3)	O6—C3'—C2'	117.7 (3)
C2—C3—C4	121.4 (3)	C4'—C3'—C2'	120.4 (3)
C2—C3—H3A	119.3	O7—C4'—C5'	123.0 (3)
C4—C3—H3A	119.3	O7—C4'—C3'	117.3 (3)
O5—C4—C10	121.5 (3)	C5'—C4'—C3'	119.6 (3)
O5—C4—C3	123.1 (3)	O8—C5'—C4'	115.8 (3)
C10—C4—C3	115.5 (3)	O8—C5'—C6'	123.6 (3)
O4—C5—C6	120.7 (3)	C4'—C5'—C6'	120.6 (3)
O4—C5—C10	119.9 (3)	C5'—C6'—C1'	119.0 (3)
C6—C5—C10	119.4 (3)	C5'—C6'—H6'A	120.5
O3—C6—C5	121.4 (3)	C1'—C6'—H6'A	120.5
O3—C6—C7	118.6 (3)	O3—C11—H11A	109.5
C5—C6—C7	120.0 (3)	O3—C11—H11B	109.5
O2—C7—C8	117.1 (3)	H11A—C11—H11B	109.5
O2—C7—C6	121.3 (3)	O3—C11—H11C	109.5
C8—C7—C6	121.6 (3)	H11A—C11—H11C	109.5
C9—C8—C7	117.7 (3)	H11B—C11—H11C	109.5
C9—C8—H8A	121.1	O7—C12—H12A	109.5
C7—C8—H8A	121.1	O7—C12—H12B	109.5
C8—C9—O1	116.3 (3)	H12A—C12—H12B	109.5
C8—C9—C10	123.6 (3)	O7—C12—H12C	109.5
O1—C9—C10	120.1 (3)	H12A—C12—H12C	109.5
C9—C10—C5	117.7 (3)	H12B—C12—H12C	109.5
C9—C10—C4	120.5 (3)	O8—C13—H13A	109.5
C5—C10—C4	121.7 (3)	O8—C13—H13B	109.5
C5'—O8—C13	119.7 (3)	H13A—C13—H13B	109.5
C4'—O7—C12	115.0 (3)	O8—C13—H13C	109.5
C3'—O6—H6A	109.5	H13A—C13—H13C	109.5
C2'—C1'—C6'	120.4 (3)	H13B—C13—H13C	109.5

### Hydrogen-bond geometry (Å, º)

**Table:**

*D*—H···*A*	*D*—H	H···*A*	*D*···*A*	*D*—H···*A*
O2—H2*A*···O3	0.82	2.38	2.762 (4)	109
O2—H2*A*···O5^i^	0.82	1.96	2.713 (4)	152
O4—H4*A*···O5	0.82	1.85	2.579 (4)	148
O6—H6*A*···O4^ii^	0.82	2.20	2.954 (3)	153
O6—H6*A*···O7	0.82	2.31	2.734 (4)	113
C2′—H2′*A*···O1	0.93	2.32	2.667 (4)	101
C3—H3*A*···O2^iii^	0.93	2.58	3.246 (5)	129
C12—H12*B*···O4^iv^	0.96	2.55	3.459 (6)	157
C12—H12*C*···O8	0.96	2.32	2.927 (6)	122

Symmetry codes: (i) *x*, −*y*+1, *z*+1/2; (ii) *x*+1, *y*−1, *z*; (iii) *x*, −*y*+1, *z*−1/2; (iv) *x*+1, *y*, *z*.
